# Happiness and Moral Courage Among Iranian Nurses During the COVID-19 Pandemic: The Role of Workplace Social Capital

**DOI:** 10.3389/fpsyt.2022.844901

**Published:** 2022-05-27

**Authors:** Maryam Pirdelkhosh, Hossein Mohsenipouya, Nouraddin Mousavinasab, Alireza Sangani, Mohammed A. Mamun

**Affiliations:** ^1^Faculty of Nursing, Mazandaran University of Medical Sciences, Sari, Iran; ^2^Health Education and Promotion, Faculty of Nursing, Mazandaran University of Medical Sciences, Sari, Iran; ^3^Department of Biostatistics, Faculty of Health, Mazandaran University of Medical Sciences, Sari, Iran; ^4^Department of Cultural Psychopathology, Farabi, Psychological Sciences Research Center, Mazandaran, Iran; ^5^CHINTA Research Bangladesh, Dhaka, Bangladesh; ^6^Department of Public Health, Daffodil International University, Dhaka, Bangladesh; ^7^Department of Public Health and Informatics, Jahangirnagar University, Dhaka, Bangladesh

**Keywords:** clinical nurses, COVID-19 pandemic, happiness, moral courage, workplace social capital

## Abstract

**Background:**

COVID-19 has become a major global health problem, and healthcare professionals are facing lot of pressure and stress. Accumulated resources and energy obtained via interpersonal relationships is called social capital, which can reduce the negative effects of pressure and stress related to the workplace by impacting happiness and moral courage. This study explored the effect of workplace social capital on moral courage and happiness in nurses working in the COVID-19 wards.

**Methods:**

In this cross-sectional study, using a random sampling method, 169 nurses from three hospitals in East Mazandaran province, Iran, participated who worked in COVID-19 wards. The Onyx and Bullen Social Capital Questionnaire, the Sekerka's Moral Courage Scale, and the Oxford Happiness Inventory were used in this study. Descriptive analysis, Pearson correlation analyses, and stepwise multiple regression were performed for data analysis.

**Results:**

The mean age of nurses was 31.38 ± 6.82 years. Socio-demographic factors such as age, gender, educational level, and employment status were significant predictors of workplace social capital. Social capital was positively correlated with moral courage (r = 0.29, *p* < 0.01) and happiness (r = 0.32, *p* < 0.01). In addition, social capital explained 6.8 and 8.6% variance in predicting moral courage and happiness, respectively.

**Conclusions:**

Workplace social capital is a vital organizational phenomenon affecting nurses' moral courage and happiness, especially during the COVID-19 pandemic. Thus, hospitals should be aware of the importance of social capital; they should ensure that all the practices and policies are in place to develop and increase it.

## Introduction

Nowadays, the Coronavirus Disease 2019 (COVID-19) has become a global public health problem. The World Health Organization announced the disease as a pandemic on March 11, 2020. In Iran, the first case of COVID was reported in early March 2019, and until October 5, 2021, more than 5.97 M Iranian individuals have been infected with the virus, and 127K deaths.

Healthcare workers (HCWs) are the frontline fighter of the pandemic. According to nurses' vital role in taking care of patients during epidemics in terms of prevention and disease control, their health is paramount to managing and overcoming infectious diseases (1). However, due to possible exposure, they are susceptible to COVID-19 infection, making them a reservoir of the virus transmission to others (2, 3). Like other healthcare workers, tremendous pressure and stress related to the workplace in their daily job is seen among the nursing staff, especially due to a shortage of personal protective equipment, inadequate support, and a high risk of infection (4–7). It is evident and consistent with the prior outbreaks that HCWs are at higher risk of developing mental health problems (8). Altered mental states among nursing professionals can impact their job satisfaction, which reduces their professional capacity (9). In addition, such a situation negatively affects their attitudes toward patients, which may appear by the changes in nursing treatment and caring behaviors (10, 11).

Accumulated energy and resources from interpersonal relationships are called social capital; it is beneficial to reduce anxiety and pressure and helps in mental stability through increasing social support and enhancing coping capacity (12–15). Thus, more investment in social capital is one of the factors associated with more social support, resulting in acquiring support from superiors, increasing the sense of belonging, and decreasing the isolation feeling (12, 16). Furthermore, the extent of social relations can be observed in the workplace, whereas the networks and interconnections in performing their duties and responsibilities make up the core of social capital (17, 18). In short, social capital is constructive in the workplace, which improves physical and mental health and increases job satisfaction in nurses (19, 20).

The experience of conflicting ethical values among the HCWs is frequent, which become prevalent during the COVID-19 pandemic. Of these ethical problems and achieving moral goals of nursing care services, patients' optimal well-being is prioritized. In order to solve ethical conflicts and fulfill moral quests related to their profession, moral courage is yet to achieve (21). The ethical problems and moral distress should not be continued forever (22–24) as this can cause adverse consequences such as distancing the nurse from nursing care and even leaving the profession for good (25, 26). In addition, irritability, burnout, sleep, and even sleep disorders can result from moral distress (27), which negatively affects their happiness and patient care quality.

Happiness is one of the indicators that reflects how any professional staff is enjoying the job (2). In the occupational nursing condition, altruism, self-confidence, obligation, creativity, kindness, and energy are required, all of which are related to happiness (28–31). Happy nurses enjoy their professional roles and responsibilities, as happiness makes them creative to perform their work and positively affects their organizational performance (32), which significantly impacts nursing services and patient satisfaction. A specific characteristic of social capital is that, in contrast to other factors, the resource does not lie in the social actors themselves (such as human capital) or the physical means of production (physical capital), but rather in the structure of relationships (33). When members of a collective perceive close relationships, they are more likely to help each other, create effective procedures, and share key information (33).

During the COVID-19 pandemic, there is a lack of studies investigating the level of moral courage and happiness among the HCWs. For instance, a Chinese study reported the changes in happiness over 6 weeks (29). A high level of moral courage (473.33 ± 1.64; range: 102–510) was found among Iranian nurses, where about 65% of the variance in moral courage was explained by age, working experience, employment status, moral sensitivity, and safe nursing care (28). Despite these findings, no studies focused on social capital and its effect on happiness and moral courage during the COVID-19 pandemic. It is hypothesized that workplace social capital might have a positive effect on moral courage and happiness, which has been investigated in this study. In addition, the predictive factors of social capital were also explored in the Iranian nurses working in the COVID-19 wards.

## Methods

### Study Site and Population

The present cross-sectional survey was conducted from April to July 2021 among the Iranian hospital nurses working in the COVID-19 wards. Utilizing a random sampling method, 169 participants were selected from 560 nurses working in three hospitals in the east of Mazandaran province affiliated with Mazandaran University of Medical Sciences, Iran. The inclusion criteria for participation in this study included: (a) registered nurses, (b) nurses involved in COVID-19 wards, and (c) voluntary participation; whereas nurses were excluded if they were (a) lactating nursing professionals (b) nurses involved in emergency work in the ward. Lactating nurses might be worried and tensed for their babies because of lack of baby care due to overwhelming duty time and fear of COVID-19 transmission to the baby.

The sample size according to the population size was estimated based on the following equation and values of σ = 1.50, d^2^ = 0.341, α = 1.96, power = 0.90, equivalent to 169 people.


$$\begin{array}{l} n=\frac{2\sigma^{2}{\left(Z_{1}-\frac{\alpha }{2}+Z_{1}-\beta \right)}^{2}}{d^{2}}=\frac{2{\left(1.50\right)}^{2}{\left(1.96+1.62\right)}^{2}}{0.341}\\ \;=169.131 \end{array}$$

### Data Collection Procedure

After adhering to the inclusion and exclusion criteria, prospective participants were conducted for data collection by two well-trained investigators. Before the investigation, each participant was informed about the study goals, where they were assured of their right to refuse to continue the survey. The informed consent was signed and returned to the researchers through e-mail; after returning informed consent, the questionnaire was also anonymously sent to the participants via the online survey. The average time to complete the data collection was almost 45 min.

### Ethical Considerations

Ethical considerations were followed in conducting this study as per the Declaration of Helsinki. After approval by the ethics committee at the Mazandaran University of Medical Sciences, two trained researchers explained the study's purpose and methods to all participants. Then, the participants signed the informed consent before partaking in the questionnaire completion.

### Measurements

#### Sociodemographic Characteristics

The basic socio-demographic characteristics such as age, gender, educational level, marital status, work experience, working section type, and personal and familial history of COVID-19 disease; were collected in this study.

#### Social Capital Questionnaire

Onyx and Bolen developed the Social Capital Questionnaire in 2000 (34). The purpose of which is to determine social capital from individuals' points of view. It has a total of 36 items compromising with eight subscales, that is, (i) Value of Life (3 items), (ii) Tolerance of Diversity (3 items), (iii) Neighborhood Connections (5 items), (iv) Family and Friends Connections (3 items), (v) Work Connections (4 items), (vi) Community Participation (7 items), (vii) Feelings of Trust and Safety (5 items), and (viii) Proactivity (6 items). The item responses were recorded on a 4-point Likert scale ranging from 1 (no, not much or no, not at all) to 4 (yes, definitely or yes, frequently). The minimum and maximum scores ranged from 36 to 144. Iranian researchers have translated this tool and the translated versions have sufficient and necessary credibility and reliability (34, 35). The validity and reliability were assessed in this study (Cronbach's alpha coefficient was 0.90).

#### Moral Courage Questionnaire

The Moral Courage Questionnaire was designed by Sekerka et al. (36). This questionnaire contains 15 questions on five aspects of (i) Moral Agency, (ii) Multiple Values, (iii) Endurance of threat, (iv) Going beyond compliance, and (v) Moral Goals. The item responses were recorded on a 7 Likert scale ranging from options from “never correct = 1” to “always correct = 7” was applied; a negative score was given for the reverse question (37). The minimum and maximum scores ranged from 15 to 105. Iranian researchers have translated this tool and the translated versions have sufficient and necessary credibility and reliability (38, 39). The validity and reliability were assessed in this study (Cronbach's alpha coefficient was 0.89).

#### Oxford Happiness Inventory

Argyle and Lu introduced this instrument; it is one of the most famous tools for self-evaluation, which has been used in most studies for assessing happiness (40). This scale consists of 29 items, where responses are recorded with a four-point Likert scale (0 = Never, 1 = Rarely, 2 = Sometimes, 3 = Always) (40). The minimum and maximum scores ranged from 0 to 87. The Oxford Happiness Inventory has achieved a high alpha coefficient of 0.94 (40). Iranian researchers have translated this tool and the translated versions have sufficient and necessary credibility and reliability (41, 42). The validity and reliability were assessed in this study (the Cronbach's alpha coefficient was 0.93).

### Data Analysis

The Kolmogorov-Smirnov test was performed to determine the distribution of data. Further, the Pearson correlation coefficient was used to determine the relationship between social capital, moral courage, and happiness. In addition, stepwise multiple regression was used to predict the level of (i) moral courage and happiness based on social capital and (ii) social capital based on demographic variables. All tests were analyzed through the SPSS statistics software version 21 at the significance level of <0.05.

## Results

### Distribution of the Socio-Demographic Characteristics

A total of 169 participants were included in this study. The mean age of 169 nurses was 31.38 (±6.823) years. About 81.1% of the participants were female, and 18.9% were male. In terms of marital status, 30.2% were single, whereas 69.8% were married, and 87.6% had a bachelor's degree.

### Mean Distributions of Social Capital, Moral Courage, and Happiness

The dispersion indices, including mean, SD, minimum, and maximum, are shown in Table 1. The results of the social capital test showed that nurses had a moderate level of social capital (86.26 ± 9.34). The mean score of moral courage was 90.24 ± 12.74, and the moral agency had a higher average score (18.21 ± 3.75) in comparison to other dimensions of moral courage. The mean score of happiness was 63.28 ± 4.78 (Table 1).

**Table 1:** Descriptive statistics of the research variables.

**Variable**	**Mean ±SD**	**Minimum score**	**Maximum score**
**Age**	31.83 ± 6.82	22	52
**Work experience** (years)	8.67 ± 3.53	2	20
**Social Capital**	86.26 ± 9.34	38	136
Community Participation	12.54 ± 4.52	7	20
Proactivity	15.08 ± 2.37	7	24
Feelings of Trust and Safety	12.69 ± 2.14	5	20
Neighborhood Connections	11.54 ± 2.07	5	19
Family and Friends Connections	7.38 ± 1.42	3	12
Tolerance of diversity	4.21 ± 0.58	2	7
Value of life	3.92 ± 0.63	2	8
Work Connections	7.30 ± 1.76	3	11
**Moral courage**	90.24 ± 12.74	15	103
Moral agency	18.21 ± 3.75	3	14
Multiple values	16.67 ± 3.06	3	15
Endurance of threats	17.79 ± 3.52	4	15
Going beyond compliance	16.40 ± 3.34	3	15
Moral goals	16.75 ± 3.21	3	14
**Happiness**	63.28 ± 4.78	30	82

### Mean Comparisons of Social Capital, Moral Courage, and Happiness

Table 2 shows the comparison of the average social capital, moral courage, and happiness of nurses based on the studied variables. Gender was significantly differed with social capital and happiness; that is, male nurses had higher scores of social capital (88.37 ± 8.55 vs. 84.16 ± 8.21; *p* = 0.007) and happiness (66.44 ± 4.90 vs. 60.13 ± 4.54; *p* = 0.009), compared to female participants. In addition, those nurses who had a master's degree were more likely to have a higher score of social capital (t=3.699, *p* = 0.001) and moral courage (t=2.564, *p* = 0.005) than bachelor nurses. In addition, the type of employment was significantly associated with social capital, moral courage, and happiness (Table 2).

**Table 2:** Mean Comparisons of Social Capital, Moral Courage, and Happiness.

**Variables**	**Category**	**N**	**Social capital**	**Moral courage**	**Happiness**
			**M (SD)**	**M (SD)**	**M (SD)**
**Gender**	Male	32	88.37 (8.55)	93.59 (11.43)	66.44 (4.90)
	Female	137	84.16 (8.21)	78.89 (10.65)	60.13 (4.54)
*t*			*2.721*	*0.561*	*2.657*
*p-value*			*0.007*	*0.575*	*0.009*
**Marital status**	Married	118	87.23 (10.59)	92.51 (10.63)	64.24 (8.34)
	Single	51	85.84 (9.80)	89.24 (9.20)	62.86 (8.21)
*t*			*.479*	*.917*	*.701*
*p-value*			*.632*	*.361*	*.484*
**Educational level**	Bachelor	148	86.08 (10.54)	90.31 (10.76)	60.69 (10.58)
	Master's	21	89.59 (11.44)	92.67 (11.43)	63.42 (12.96)
*t*			*3.699*	*2.564*	*.674*
*p-value*			* <0.001*	*.005*	*.234*
**Employment type**	Temporary official	30	87.48 (10.93)	90.06 (10.34)	65.84 (11.46)
	Official	39	86.34 (11.54)	88.99 (12.08)	62.76 (10.22)
	Company	31	89.08 (9.80)	96.90 (10.72)	64.29 (12.03)
	Design	53	78.04 (9.24)	84.25 (11.66)	58.43 (11.89)
	Contractual	16	85.70 (10.64)	90.55 (9.51)	61.12 (10.65)
*F*			*3.255*	*2.980*	*3.667*
*p-value*			* <0.001*	* <0.001*	* <0.001*
**COVID-19 infection**	Yes	85	85.54 (11.21)	87.50 (10.22)	62.81 (10.04)
	No	84	86.99 (11.78)	92.97 (11.09)	63.75 (10.43)
*t*			*.539*	*1.671*	*.520*
*p-value*			*.590*	*.097*	*.604*
**Section type**	Emergency	35	62.20(10.7)	79.65(14.4)	68.05(13.5)
	CCU	25	60.94(10.8)	84.42(10.7)	65.72(11.7)
	ICU	22	61.04(9.2)	81.75(11.5)	64.10(10.3)
	Surgery room	17	53.18(9.6)	78.51(14.1)	59.22(10.7)
	Internal	16	53.16(6.9)	82.08(11.6)	62.01(10.6)
	Surgery	15	61.43(10.4)	81.51(11.8)	68.50(13.3)
	Children	9	60.95(16.2)	84.59(11.0)	6.99(9.4)
	Other	30	61.02(7.6)	87.06(8.5)	67.95(11.1)
*F*			*1.613*	*1.348*	*1.505*
*p-value*			*0.135*	*0.231*	*0.169*

### Pearsons Correlation Coefficient

**Figure 1:**
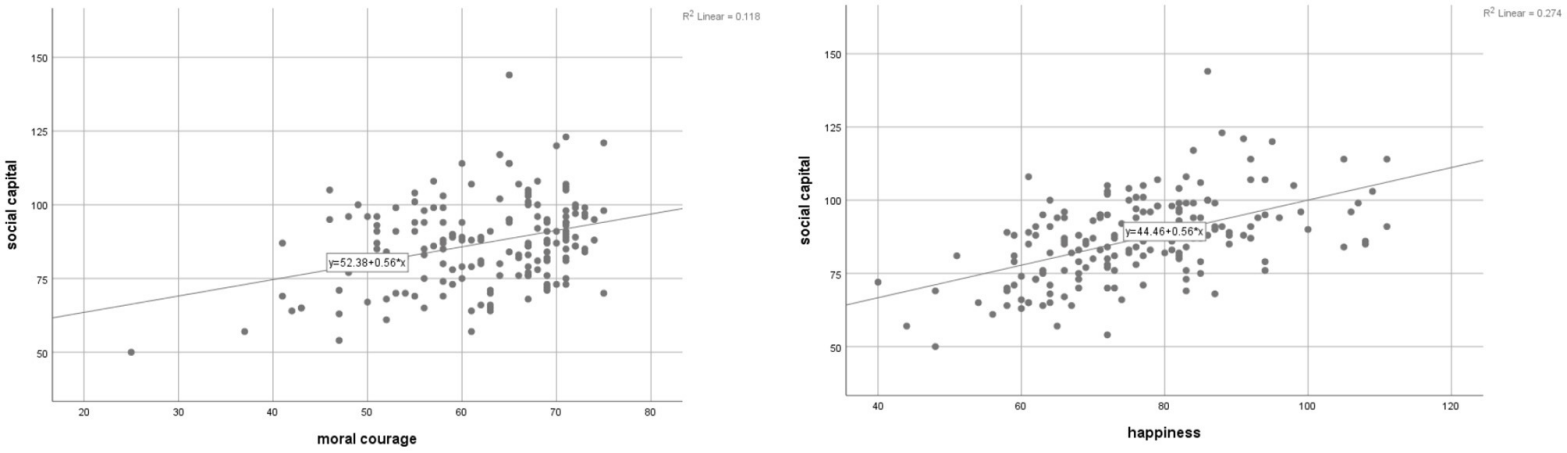
Correlation between **(A)** social capital and moral courage; and **(B)** social capital and happiness.

The results of the correlation analyses are shown in Table 3. A positive correlation was identified between social capital, moral courage, and happiness (*p* < 0.001), that is, higher social capital was positively associated with higher moral courage and happiness (Figure 1).

**Table 3:** Correlation coefficient of the continuous variables.

**Variable**	**Social Capital**	**Moral Courage**	**Happiness**
Social Capital	1		
Moral Courage	0.29^**^	1	
Happiness	0.32^**^	0.36^**^	1
Age	0.21^**^	0.15^*^	0.18^*^

^**^p < 0.01 ^*^p < 0.05.

### Stepwise Regression Analyses

The results in Table 4 predict moral courage based on social capital. Social capital significantly predict moral courage. Result revealed that social capital can explain 6.8% variance in predicting moral courage. Additionally, the regression coefficient (beta) suggested that for every 1 unit increase in social capital the moral courage will increase by 0.225 units.

**Table 4:** Regression and effect coefficients of social capital in moral courage.

**Predictive variables**	**R**	**[Beta]**	***R* ^2^**	**S E**	**t**	**Sig**.
Social Capital	0.261	0.225	0.068	2.142	2.887	<0.001

The results in Table 5 predict happiness based on social capital. Social capital significantly predict happiness. Result revealed that social capital can explain 8.6% variance in predicting moral courage. Additionally, the regression coefficient (beta) suggested that for every 1 unit increase in social capital the moral courage will increase by 0.253 units.

**Table 5:** Regression and effect coefficients of social capital in happiness.

**Predictive variables**	**R**	**[Beta]**	***R* ^2^**	**SE**	**t**	**Sig**.
Social Capital	0.294	0.253	0.086	2.355	3.453	<0.001

The results in Table 6, predict social capital based on socio-demographic variables. It is found that all of the factors (age, gender, educational level, and employment status) were significant predictors of social capital.

**Table 6:** Regression and effect coefficients of socio-demographic variables in social capital.

**Predictive variables**	**R**	**[Beta]**	***R* ^2^**	**SE**	**t**	**Sig**.
Age	0.182	0.134	0.033	2.443	2.331	0.027
Sex	0.167	0.119	0.027	2.867	2.054	0.043
Educational level	0.254	0.237	0.064	2.121	2.744	0.005
Employment type	0.219	0.182	0.047	2.533	2.471	0.014

## Discussion

This study is the first approach to investing the relationships between workplace social capital and happiness and moral courage among nurses engaged in treating the COVID-19 patients. It is observed that the workplace social capital influences nurses' happiness and moral courage during the COVID-19 pandemic.

Social capital is being reported to be associated with common socio-demographic factors. For instance, Saberi et al. (43) found that gender had a significant association with trust and security from the dimensions of social capital, where women had lower trust and security scores than men. As consistent with that study, gender was found as the significant predictor of social capital in this study. In addition, evidence suggests that the oldest age people have the highest level of trust, and those with the highest education tend to have more social capital (44). In the present study, people with higher literacy levels were higher in terms of social capital. The independent and joint effects of three dimensions of demographic diversity (gender, race, and age) on organizational social capital in the US federal government were also reported, where there was a negative relationship between age diversity and organizational social capital (45). However, two elements of social capital, including the value of life and feelings of safety and trust, were reported as the consistent predictors of health, life satisfaction, and happiness (46). At the same time, the results of Kim et al.'s study (47) found that among social capital factors, two elements of feelings of trust and safety, neighborhood connections had a significant and positive mediating role in the relationship between sports participation and general happiness. Such a positive association between workplace social capital and happiness has also been proved in European countries, for example, the results of the RodrguezPose's study (48). Furthermore, Hosseinbor and Nabizadeh's study (49) also indicated a positive and significant relationship between social capital with happiness among Iranian medical school faculty members in that country; the present study was also carried out.

Social capital and social support increment were found to have resiliency against COVID-19 with the mediating role of spiritual happiness (50). A person with a high level of happiness is more satisfied with their life because they experience more positive emotions. A high level of workplace social capital explains established extensive contact between nurses and other workplace personnel through long-term interaction and exchange (18). A strong sense of responsibility, identity, and social norms can be obtained by enriching and strengthening network connections among colleagues (51). During social interactions, nurses are likely to encounter emotional behaviors from leaders and colleagues, which helps build up a healthy interpersonal relationships and spreads positive senses (52). Therefore, some factors such as a pleasant workplace atmosphere and good communications with leaders, colleagues, and patients can reduce the work pressure and stress among nurses; they can also attenuate stress impress professional identity (53). Thus, health policy-makers and authorities can make some interventions to develop strategies for improving nurses' happiness and social capital.

Researchers have recently begun to study workplace social courage's role in organizational results; they suggest that courageous behaviors affect positive work behavioral outcomes (54–58). In Mert's study (59), workplace social courage is a facilitator for subjective happiness and life satisfaction. Awareness of the importance of moral courage and its influential factors can help healthcare researchers, educators, clinicians, and leaders demonstrate moral courage, face ethical challenges and ethical environment maintenance (36). It is essential that HCWs value and support their counterparts who dare to resist and converse against unethical behaviors. Professional nursing organizations should countenance nurses to do measures that create and sustain ethical environments; they also should support protections for individuals who combat unethical behaviors in the workplace. All educational and continuing education programs should elevate moral courage; the educational content should include strategies that enable nurses to act bravely when ethical standards are neglected (60).

One of the study's limitations was filling in the questionnaire in the challenging conditions of the nurses, who tried to overcome this by providing the questionnaire online. Another limitation was the self-reported nature of the study, which may have received more positive responses. In order to overcome the limitation, an attempt was made to give the company the necessary assurance that the information was confidential. It should be noted that many factors associated with the dependent variables were not assessed in this study, which can be considered in further studies. Finally, results from cross-sectional data limit the causal interpretation of the associations.

## Conclusions

Social capital is an important organizational event, influencing organizational results, including happiness and moral courage among nurses, especially in the clinical settings during the COVID-19 pandemic, as found in this study. Based on the current study's findings, it can be stated that social capital is an essential construct for hospital upgrading. Thus, hospitals need to consider the importance of social capital and organize all policies and practices to promote and enhance social capital. They also should understand the dynamics of social capital and remove all the barriers to social capital development. Furthermore, there should be specialized institutions in universities and official institutions in charge of identifying this concept and planning to promote it, and then monitoring the concept of social capital. Also, the media can do a lot to help make social capital more transparent and recognizable.

## Data Availability Statement

The raw data supporting the conclusions of this article will be made available by the authors, without undue reservation.

## Ethics Statement

The studies involving human participants were reviewed and approved by Mazandaran University of Medical Sciences (Approval No. IR.MAZUMS.REC.1399.8950). The patients/participants provided their written informed consent to participate in this study.

## Author Contributions

MP, HM, NM, and AS conceptualized the study, collected data, and interpreted the results. MM reanalyzed the data, reviewed, and edited the draft extensively. All authors contributed to the writing of the manuscript, approved the final version of the manuscript, and agreed to be accountable for all aspects of the work.

## Conflict of Interest

The authors declare that the research was conducted in the absence of any commercial or financial relationships that could be construed as a potential conflict of interest.

## Publisher's Note

All claims expressed in this article are solely those of the authors and do not necessarily represent those of their affiliated organizations, or those of the publisher, the editors and the reviewers. Any product that may be evaluated in this article, or claim that may be made by its manufacturer, is not guaranteed or endorsed by the publisher.
